# Resembling Acute Bright’s Disease

**Published:** 1884-10

**Authors:** J. T. Kent


### RESEMBLING ACUTE BRIGHT'S DISEASE.

Child of C. N., a sprightly little girl two years old: Symptoms, yellowish white discharge from left ear, profuse lacrymation from right eye, and some white, clear mucus flowing over the eye-lid and cheek that seemed to be blood. The under lid of right eye appeared like a water-bag, and the whole face was puffed and rather transparent; the right was much the worse of the two. The child looked sickly and cried much. The mother stated that it had not been well for several weeks. While thinking over Apis and Ars. I began to look for agg. by warmth, etc., when I was informed that the child could not endure any covering, and she was absolutely thirstless, which excluded Arsenic from further consideration. The mother believed the urine to be scanty but she was not certain, but says she, "Her water smells strong, like that of a horse." Nit-ac.$^{1200}$ (Jenichen), and we soon cured the case, ear discharge and all, in the surprisingly short time of a few days.

This was not a case of *acute Bright's disease* following scarlet fever, but much like it. Every physician living in this malarial climate must have observed the same anasarca and otorrhœa following malarial attacks, home-treated or neglected. The dropsical condition follows all kinds of malarial attacks, and particularly this ear complication, associated with kidney disease. But will some astute pathologist inform me why nitric acid cured this case so promptly if on other grounds than that it was the *simillimum?* These cases all die when not properly treated! They all recover promptly on a few doses of the appropriate remedy.—J. T. KENT.

